# Uptake of CCR7 by KIR2DS4^+^ NK Cells Is Induced upon Recognition of Certain HLA-C Alleles

**DOI:** 10.1155/2015/754373

**Published:** 2015-04-16

**Authors:** Silvia Pesce, Simona Carlomagno, Alessandro Moretta, Simona Sivori, Emanuela Marcenaro

**Affiliations:** ^1^Dipartimento di Medicina Sperimentale, Università degli Studi di Genova, Sezione di Istologia, Via G.B. Marsano 10, 16132 Genova, Italy; ^2^Centro di Eccellenza per le Ricerche Biomediche, Università degli Studi di Genova, Viale Benedetto XV 9, 16132 Genova, Italy

## Abstract

The KIR2DS4 receptor is the oldest KIR2DS expressed by human NK lymphocytes. The specificity of recognition of this receptor for various HLA class I alleles has been demonstrated; however it remains poorly understood whether these interactions may result in the activation of some specific functions in NK cells. Here, we examined the functional outcome of the KIR2DS4/HLA class I interaction by the use of an alternative functional system based on the ability of KIR2DS4 to regulate the mechanism of trogocytosis by NK cells. We demonstrate that KIR2DS4 can induce the uptake of CCR7 by KIR2DS4^+^ NKG2A^+^ NK cell clones after interacting with CCR7^+^ target cells expressing HLA-Cw4 and HLA-Cw6 alleles. However this interaction is not always sufficient to override the inhibition generated by NKG2A expressed on the same NK cells. The recognition of HLA-Cw4 was confirmed by experiments of cytotoxicity against HLA-C-transfected cells. We also show that, different from resting NK cells, the acquisition of CCR7 in response to IL-18 cannot occur in IL2-activated NK cells because of a marked downregulation in their IL-18R*α* expression. As a consequence trogocytosis represents the major mechanism by which KIR2DS4^+^ activated NK cells acquire the expression of this chemokine receptor.

## 1. Introduction

NK cells are tuned by a set of cell surface receptors that finely regulate their effector functions against cancer cells and infected cells [1–3]. These receptors include the lectin-like heterodimers CD94/NKG2C (activating form) and CD94/NKG2A (inhibitory form), specific for HLA-E, a nonclassical MHC molecule characterized by a limited polymorphism [4, 5], and the killer cell immunoglobulin-like receptors (KIRs) [6–11]. KIR molecules have been shown to be key factors that influence the NK-mediated control of at least some tumours or viral infections. The KIR family includes both inhibitory and activating KIRs. The inhibitory ones (KIR2DL and KIR3DL) are nonrearranged HLA class I-binding receptors, able to distinguish among different HLA-C, -B, and -A allotypes [6]. The activating ones include KIR2DS1, KIR2DS2, KIR2DS3, KIR2DS4, KIR2DS5, and KIR3DS1, whose ligands and functions in immune response remain poorly understood and still enigmatic. The main differences between inhibitory and activating KIRs are located in their cytoplasmic tails. Indeed, the activating KIRs are characterized by a short cytoplasmic tail lacking ITIMs and by a transmembrane domain with a charged amino-acid residue that enables association with ITAM-bearing molecules [6, 12].

Despite the fact that the extracellular domains of activating KIRs are highly homologous to their inhibitory counterparts, only for some of them the specificity for HLA class I molecules has been demonstrated. In particular KIR2DS1 recognizes HLA-C2 alleles and KIR2DS4 binds to HLA-A^∗^1102 and to a limited number of HLA-C1/-C2 alleles (three with C1-epitope: C^∗^ 1601, C^∗^ 0102, and C^∗^ 1402, and three with C2-epitope: C^∗^ 0501, C^∗^ 0202, and C^∗^ 0401), whereas KIR3DS1^∗^014 binds to HLA-Bw4 alleles [11–17].

The KIR gene-cluster is divided into group A haplotypes, dominated by inhibitory KIRs, and group B haplotypes that, in addition to a varying number of inhibitory KIRs, contain up to five activating KIRs [9, 18, 19]. Remarkably, KIR2DS4 is the only activating KIR present in A haplotypes [18, 20].

The interactions of variable KIRs with polymorphic HLA class I ligands form an extraordinary immunogenetic system that influences NK cell biology, human susceptibility to disease, and the success of hematopoietic cell transplantation (HCT) [3, 21].

Different studies have suggested that the activating KIRs could interact with HLA class I, but at a lower affinity than their inhibitory counterparts. However, during viral infections, their HLA affinity may be heightened by the presentation of viral peptides, enabling NK-mediated killing of infected cells [22]. Thus, similar to T cells, also NK cell responses may be conditioned by the nature of the HLA class I presented peptide [23]. In this context, KIR2DS1 differently binds to HLA-Cw4 depending on the type of peptide associated [14].

It has been shown that infection with human Cytomegalovirus may induce expansion of NK cells expressing activating KIRs, including KIR2DS4, KIR2DS2, or KIR3DS1 [24], even independently of the expression of NKG2C [25, 26].

In addition, several reports suggest that viral infections (including HCV and HIV) are, at least in part, controlled by activating KIRs [27–29], even if in recent reports a role for KIR2DS4 has been proposed in promoting HIV-1 pathogenesis during chronic infection [30, 31].

Finally, it is conceivable that the activating KIRs can also recognize non-HLA class I ligands. In this context, it has been described that KIR2DS4 is able to interact with a protein expressed on melanoma cell lines and on a primary melanoma [32].

Recently, the potential value of alloreactive NK cells expressing activating KIRs in HCT has been demonstrated [33–36]. In this context, Cooley et al. found that clinical outcome of HCT from an unrelated donor (as therapy for acute myelogenous leukemia) was improved when the donors have one or two KIR B haplotypes (KIR B/x donors) compared to donors who have two KIR A haplotypes (KIR A/A donors) [37].

Moreover our previous data suggest that in KIR/KIR-ligand mismatched haplo-HCT a remarkable advantage may exist in selecting KIR2DS1^+^ donors to be used in C2^+^ recipients not only for their killing capability against recipient's leukemic cells, dendritic cells (DCs), and T cell blasts but also for the induction of CCR7 uptake from both allogeneic DCs and T cell blasts themselves [33, 34, 36]. Notably this mechanism may be relevant for turning KIR^+^ NK cells that usually do not express this chemokine receptor, into lymph nodes migrating cells [38].

Importantly, this* de novo* CCR7 expression may represent a major mechanism by which alloreactive (i.e., activated) NK cells can migrate to lymph nodes, kill recipient's DCs, and prevent priming of alloreactive donor's T cells as well as induction of graft-versus-host disease (GvHD) [36, 38].

In this study we show that an important functional outcome of the interaction between KIR2DS4^+^ NK cells and CCR7^+^ cells expressing suitable HLA-I ligands (i.e., HLA-Cw4 and to a lesser extent of HLA-Cw6) is represented by the acquisition of CCR7 by a mechanism of trogocytosis. The uptake of this chemokine receptor may have important implications for alloreactive NK cells developing in transplanted patients after HCT.

## 2. Materials and Methods

### 2.1. Antibodies

Anti-hCCR7 (IgG2a, clone 150503), anti-KIR2DL1 fluorescein isothiocyanate-conjugated (clone 143211), and anti-IL-18R*α* (IgG1) mAbs were from R&D Systems Inc. (Abingdon, United Kingdom). Anti-CD56 phycoerythrincyanine 7- (PC7-) conjugated (clone C218), anti-NKG2A allophycocyanin-conjugated (clone Z199), anti-KIR2DL2/L3 PC7-conjugated (clone GL183), and anti-CD83 (IgG2b) mAbs were from Beckman Coulter, Immunotech (Marseille, France). Anti-KIR3DL1 biotin-conjugated (clone DX9), anti-biotin Vio700, anti-KIR2DL1/S1 biotin-conjugated (clone 11PB6), and anti-biotin Vioblue mAbs were purchased from Miltenyi Biotec (Bergisch Gladbach, Germany). Anti-human HLA-E (clone 3D12) was purchased from Biolegend (San Diego, CA) and anti-HLA-C (clone C-8) was purchased from Santa Cruz Biotechnology (CA).

A6/136 and F(ab′)_2_, 6A4 (IgM and IgG1, resp., anti-HLA-I), Y9 (IgM, anti-CD94), DF200 (IgG1, anti-KIR) [39], FST172 (IgG2a, anti-KIR2DS4), and PAX180 (IgG1, anti-KIR2DS4) were produced in our laboratory. The 6A4 mAb was previously shown to affect recognition by C2-specific KIRs but not by C1-specific KIRs [40].

### 2.2. Cell Preparations and Cytofluorimetric Analysis

All biological samples were collected after obtaining informed consent and the study was approved by the ethical committee of IRCCS S. Martino-IST (39/2012). Informed consent was provided according to the Declaration of Helsinki.

PBMC from volunteer blood donors admitted at the blood transfusion center of IRCCS S. Martino-IST were derived by Ficoll-Hypaque gradients (Sigma-Aldrich, St. Louis, MO, USA) and purified by negative selection, using NK cell isolation kit II (Miltenyi Biotec GmbH, Bergisch Gladbach, Germany). The purity of NK cells was >98% as assessed by flow cytometric analysis. In order to obtain polyclonally or clonally activated NK cells, freshly isolated (resting) NK cells were cultured on irradiated feeder cells in the presence of 100 U/mL rhIL-2 (Proleukin, Chiron, Emeryville, CA) and 1,5 ng/mL phytohemagglutinin (GIBCO Ltd, Paisley, Scotland) [34].

The medium used throughout the experiments was RPMI 1640 medium supplemented with 2 mM L-glutamine, 1% penicillin-streptomycin-neomycin mixture, and 10% heat-inactivated FCS.

For trogocytosis experiments [38], NK cells were plated at 1 × 10^5^ cells/mL in round-bottom 96-well tissue culture plates (Costar, Corning Incorporated, New York, USA) in the presence of CCR7^+^ 221 cells (LCL 721.221, human Epstein-Barr virus- (EBV-) transformed B cell line), transfected or not with different HLA class I alleles, at a ratio of 1 : 1, spun down for 3 min at 800 rpm, and incubated for different time points (from 15 to 30 minutes) at 37°C in 5% CO_2_. Next, NK cells were directly assessed for surface phenotype.

In other experiments, resting (NK) or polyclonal IL-2 activated (BULK) NK cells were cultured for a short-term (18 hours) in the presence of either 0.1 *μ*g/mL of hIL-18 (Biosource International Inc.) or 0,5 ng/mL of IL-12 (Peprotech Inc., London, GB).

The 221 cell lines and the 221-Cw3 and 221-Cw4 transfectants were kindly provided by Biassoni (Istituto Giannina Gaslini, Genova, Italy) [41, 42]. The 221-Cw6 transfectant was kindly provided by Professor Falk (Institute for Immunology, University of Munich, Germany) [43]. Cell lines were kept at low passage, returning frequently to original frozen stocks. Before freezing and after defrosting the cells were always checked for their phenotype.

Phenotypic analyses were performed by flow cytometry (FACSVerse; Becton, Dickinson).

### 2.3. Cytolytic Activity of NK Cell Clones

Cells used as targets in the various cytolytic assays were represented by untransfected or HLA-Cw3, -Cw4, or -Cw6-transfected 221 cell lines. The NK-mediated cytotoxicity was assessed in a 4 h ^51^Cr release assay as previously described [34, 38]. For masking experiments, NK cells were preincubated with mAbs specific to the various NK receptors or with anti-HLA class I mAb (A6/136 or F(ab′)_2_ 6A4) 10 minutes prior to addition of target cells; mAb concentration was 10 *μ*g/mL. The* E/T* ratio used was 1 : 1.

### 2.4. Statistical Analysis

Mann-Whitney nonparametric test was employed for evaluating quantitative variables. The statistical level of significance (*p*) is indicated. Graphic representation and statistical analysis were performed using the PASW Statistics, version 20.0 software (formerly SPSS Statistics) (IBM, Milan, Italy), and GraphPad Prism 6 (GraphPad Software, La Jolla, CA).

## 3. Results and Discussion

### 3.1. KIR2DS4 Induces CCR7 Expression on NK Cell Clones after Interactions with B-EBV Cell Lines Transfected with Certain HLA-C Alleles

In light of the data recently obtained in our laboratory on the beneficial effects mediated by KIR2DS1 in the acquisition of CCR7 by licensed alloreactive KIR^+^ NK cells and of the important clinical implications that this event can determine, we evaluated whether the expression of KIR2DS4 may favor the uptake/capture of CCR7 during interaction with CCR7^+^ target cells transfected with specific HLA class I alleles.

To this aim, we generated two series of NK cell clones that were selected from two different types of individuals (one belonging to the A and the other to the B haplotype) on the basis of their KIR2DS4^+^ NKG2A^+^ or KIR2DS4^−^ NKG2A^+^ phenotype.

**Figure 1:**
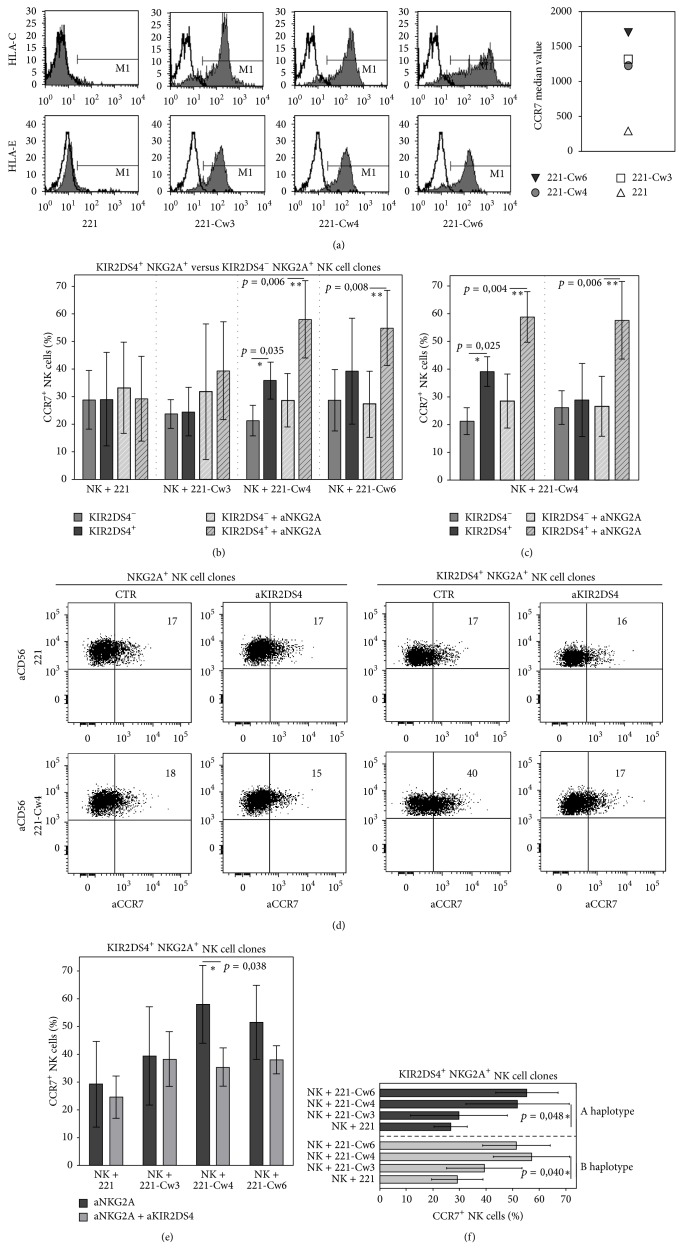
*De novo* expression of CCR7 in NK cell clones upon interaction with transfected 221 cell lines: comparison between KIR2DS4^+^ and KIR2DS4^−^ NK cell populations. (a) Expression profile of HLA-C and HLA-E molecules (left panels) and of CCR7 receptor (right panel) on 221 cells or 221 cells transfected with HLA-Cw3 or HLA-Cw4 or HLA-Cw6 is shown. (b) KIR2DS4^−^ NKG2A^+^ or KIR2DS4^+^ NKG2A^+^ NK cell clones were incubated with untransfected 221 cells or 221 cells transfected with HLA-Cw3 or HLA-Cw4 or HLA-Cw6 alleles. NK cell clones (6 for each type) were derived from one B haplotype donor. Coincubation was performed in the absence or presence of anti-NKG2A mAbs, as indicated. The histogram represents the percentage of CCR7^+^ NK cells after incubation with untransfected or HLA-transfected 221 cells. The average of 6 independent experiments and standard deviation (mean ± SD) are indicated. ^∗∗^ *p* < 0.01; ^∗^ *p* < 0.05. (c) KIR2DS4^−^ NKG2A^+^ or KIR2DS4^+^ NKG2A^+^ NK cell clones (from one B haplotype donor) were incubated with 221-Cw4 cells. 3 KIR2DS4^−^ NKG2A^+^ and 3 KIR2DS4^+^ NKG2A^+^ NK cell clones characterized by low NKG2A-mediated inhibition (on the left) and 3 KIR2DS4^−^ NKG2A^+^ and 3 KIR2DS4^+^ NKG2A^+^ characterized by high NKG2A-mediated inhibition (on the right) are represented. The average of 6 independent experiments and standard deviation (mean ± SD) are indicated. ^∗∗^ *p* < 0.01; ^∗^ *p* < 0.05. (d) A representative KIR2DS4^+^ NKG2A^+^ NK cell clone (derived from one B haplotype donor) was incubated at 37°C with CCR7^+^ untransfected 221 cells or 221 cells transfected with HLA-Cw4. After 30 minutes, NK cells were harvested and stained with anti-CD56 and anti-CCR7 mAbs for cytofluorimetric analysis. The percentages of CCR7^+^ cells are indicated in the upper-right corners. (e) KIR2DS4^+^ NKG2A^+^ NK cell clones derived from the one B haplotype donor were incubated with untransfected 221 cells or 221 cells transfected with HLA-Cw3 or HLA-Cw4 or HLA-Cw6 alleles. Coincubation was performed in the absence or in the presence of mAbs to the indicated molecules. The histogram represents the percentage of CCR7^+^ NK cells after incubation with untransfected or HLA-transfected 221 cells. The average of 6 independent experiments and standard deviation (mean ± SD) are indicated. ^∗^ *P* < 0.05. (f) KIR2DS4^+^ NKG2A^+^ NK cell clones derived from one A haplotype donor (upper panel) and one B haplotype donor (lower panel) (3 for each haplotype) were incubated with untransfected 221 cells or 221 cells transfected with HLA-Cw3 or HLA-Cw4 or HLA-Cw6 alleles. The average of 3 independent experiments and standard deviation (mean ± SD) are indicated. ^∗^ *p* < 0.05.

These NK cell clones were cocultured with the CCR7^+^ LCL 721.221 human EBV-transformed B cell line (221 cells) transfected with HLA-Cw3 (C1-epitope), or with HLA-Cw4 (C2-epitope), or with HLA-Cw6 (C2-epitope) alleles. The phenotype of these cell lines, in terms of HLA-ligands and CCR7, is shown in Figure 1(a). After short-term coincubation, cells were harvested and stained with anti-CCR7 mAb in combination with anti-CD56 (used to distinguish NK cells from 221 cell lines).

As shown in Figure 1(b), all NK cell clones expressed CCR7 after coculture in the presence of untransfected or HLA-transfected 221 cells. As previously shown, this* de novo* expression of CCR7 was due to capture of this receptor from 221 and involved the function of both NKp46 and adhesion molecules [38]. The level of CCR7 expression on KIR2DS4^+^ NK cell clones was substantially comparable to that detected on KIR2DS4^−^ NK cell clones. A remarkable exception however was represented by KIR2DS4^+^ NKG2A^+^ NK cell clones that were cocultured with Cw4-transfected cells. Thus under these conditions most (but not all) of these NK cell clones displayed higher levels of CCR7 as compared to those expressing the KIR2DS4^−^ NKG2A^+^ phenotype (Figures 1(b) and 1(c)). Importantly this effect was prevented by the presence of anti-KIR2DS4 mAb during coculture (Figures 1(d) and 1(e)) but not by other IgG2a isotype control mAbs (not shown).

Notably, after coculture with 221-Cw4 transfectants, the capability of CCR7 uptake by KIR2DS4^+^ NKG2A^+^ NK cell clones was further increased in the presence of mAb-mediated blocking of NKG2A (Figures 1(b) and 1(c)). Under these conditions, increased levels of CCR7 uptake were observed in substantially all KIR2DS4^+^ NKG2A^+^ NK cell clones analyzed (Figure 1(c)).

In addition, the disruption of the NKG2A/HLA-E interaction resulted in a significant increase of CCR7 uptake by the same clones after interaction with 221-Cw6 (Figure 1(b)). On the other hand, only a minor fraction of KIR2DS4^+^ NKG2A^+^ NK cell clones expressed CCR7 when cocultured with 221-Cw3 in the presence of anti-NKG2A mAb (Figure 1(b)). Remarkably, also the uptake of CCR7 observed in the presence of anti-NKG2A mAb (after interaction with 221-Cw4 and 221-Cw6 cell lines) could be prevented by blocking of KIR2DS4 with the specific mAb (Figure 1(e)).

Differently from KIR2DS4^+^ NKG2A^+^ NK cell clones, KIR2DS4^−^ NKG2A^+^ NK cell clones always displayed similar levels of CCR7 when cocultured with 221 cell lines transfected with either C1- or C2-epitope (Figure 1(b)).

Notably, no significant differences could be appreciated between NK cell clones derived from A and B haplotypes in the ability to recognize HLA-class I transfectants and in CCR7 uptake (Figure 1(f)).

Altogether these data indicate that, similar to KIR2DS1, the activating signals generated by KIR2DS4 after interaction with HLA-Cw4 can induce the uptake of CCR7. However a certain heterogeneity could be observed among different KIR2DS4^+^ NKG2A^+^ NK cell clones; thus in some clones the activating signal generated via KIR2DS4 could not override the inhibitory signals generated by the NKG2A/HLA-E interaction and required the presence of anti-NKG2A mAb during coculture in order to mediate CCR7 uptake (Figure 1(c)).

So far the biological role of KIR2DS4 receptor has not yet been fully clarified. It is possible that this receptor may recognize different alleles of the HLA class I but that the functional outcome of this interaction is often blurred by other (inhibitory) receptors simultaneously expressed by the same cell.

Our results suggest that, in given donor (KIR2DS4^+^)/recipient (HLA-Cw4^+^) pairs, KIR2DS4 expression may contribute to the induction of the* de novo* expression of CCR7 on the surface of activated NK cells. This event is regulated at least in some cases by inhibitory receptors/HLA class I interactions (in this case NKG2A). This uptake is particularly evident after interaction with HLA-Cw4^+^ target cells [15, 16] and to a lesser extent with HLA-Cw6. At this regard it is worth remembering that in a previous study little binding of KIR2DS4-Ig to 221-Cw6 cells could be observed [16].

### 3.2. IL-18 Induces CCR7 Expression Only on Resting NK Cells, but Not on IL-2 Activated NK Cell Population

In previous studies, Mailliard et al. [44] demonstrated that the CD56^dull^/CD16^+^ NK subset may* de novo* express CCR7, when exposed to exogenous IL-18, a proinflammatory cytokine released in inflamed tissue by innate cells (such as macrophages).

**Figure 2:**
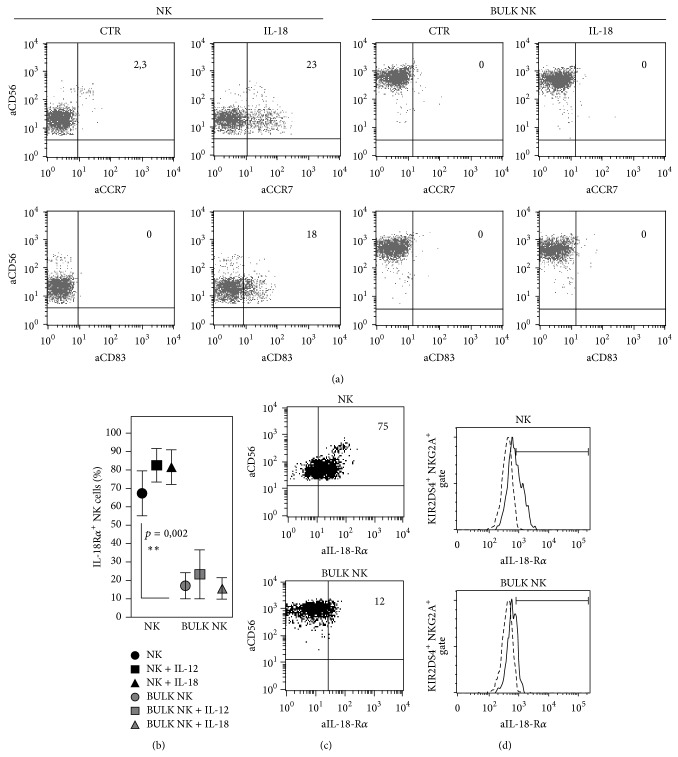
Analysis of the IL-18R*α* surface expression on resting or activated NK cells: functional implications. (a) Freshly isolated (NK) and IL-2 activated (BULK) NK cells from one B haplotype donor were cultured in the presence of exogenous IL-18 for 18 hours and then analyzed for CCR7 expression. Analysis of CD83 expression on NK cells was used as a positive control of the effect mediated by IL-18. The values reported in the upper-right corners indicate the percentage of CD56^+^ CCR7^+^ or of CD56^+^ CD83^+^ NK cells. A representative of 10 independent experiments performed using different donors is shown. (b) Freshly isolated or short-term (18 h) activated NK cells (NK) from one B haplotype donor were analyzed for the expression of IL-18R*α* in comparison with long-term IL-2 activated (BULK) NK cells form the same donor. BULK NK cells were also treated for 18 h with either IL-18 or IL-12, after washing the cells, to remove IL-2 from the supernatant and then analyzed for the expression of IL-18R*α*. The average of 6 independent experiments and standard deviation (mean ± SD) are indicated. ^∗∗^ *p* < 0.01. (c) A representative B haplotype donor is shown for the expression of IL-18R*α* on both resting and BULK NK cells. (d) The histogram plots refer to the expression of IL-18R*α* on both resting and BULK NK cells of a representative B haplotype donor after gating on KIR2DS4^+^ NK cells. The dashed line represents the isotype control.

We show that, at variance with resting NK cells, which can express CCR7 in an IL-18 dependent fashion, polyclonal IL-2 activated (BULK) NK cells as well as NK cell clones are unable to express this receptor in response to IL-18 (Figure 2(a)). This different behaviour might be explained by a different expression of IL-18 receptor alpha (IL-18R*α*) on resting as compared to activated NK cells. In particular, BULK NK cells express significantly lower levels of this receptor as compared to resting NK cells, even after short-term treatment with either IL-18 or IL-12 cytokines (Figures 2(b) and 2(c)). In contrast, short-term activation of resting NK cells with both of these cytokines induces a further upregulation of IL-18R*α* on their surface (Figure 2(b)). This difference in IL-18R*α* expression between resting and activated NK cells could also be appreciated after gating on KIR2DS4^+^ NKG2A^+^ cells (Figure 2(d)).

In humans, a number of NK cell subsets with different functional potential have been described. The least mature NK cells are represented by the CD56^bright^ CD57^−^ subset, which may give rise to the CD56^dim⁡^ CD57^−^ cells. This subset, in turn, matures into the most mature CD56^dim⁡^ CD57^+^ subset. These three steps of maturation are associated with changes in phenotypic and functional features of NK cells [45–48].

Previous reports indicated that CD57^+^ NK cells may respond to IL-18 less well than other subsets; accordingly their expression of IL-18R*α* is lower as compared with CD57^−^ NK cells [24, 46, 49].

The ability of resting NK cells to respond to exogenous cytokines is crucial for the acquisition of their full competence and correlates with their ability to control infections. In particular, IL-18 is able to induce high expression of CD25, high release of IFN-gamma (especially in combination with IL-12), and, importantly, the* de novo* expression of CCR7 by KIR^+^  CD56^dull^ NK cells [38, 44, 50, 51].

Here we show that the *α*-chain of IL-18 receptor is expressed by both circulating CD56^bright^ and CD56^dull^ NK cell subsets (although at higher levels on the CD56^bright^ subset) (Figures 2(b) and 2(c)) and upregulated after short-time activation with either IL-18 or IL-12 (Figure 2(b)).

Remarkably, however, this receptor is downmodulated in polyclonal IL-2 activated (BULK) NK cells (Figures 2(b) and 2(c)). Moreover, treatment of BULK NK cells with either IL-18 or IL-12 could not induce IL-18R*α* upregulation (Figure 2(b)).

IL-18-mediated CCR7 expression is an important event to allow the migration of CD56^dull^ NK cells towards lymph nodes, where this subset may exert their regulatory role in respect to adaptive immune responses [44, 52]. However, our results suggest that activated NK cells, including NK cell clones, are unable to upregulate CCR7 in response to IL-18 because of the loss of the specific receptor for this cytokine.

### 3.3. KIR2DS4 Represents an Advantage in the Killing of HLA-Cw4^+^ Target Cells

The same NK cells used for trogocytosis experiments were assessed for cytolytic activity induced upon interaction with 221 cells transfected or not with different HLA class I molecules. In this context, it is important to remember that the transfer of target cell molecules to NK cells does not appear to require killing of target cells as suggested, for instance, by the time required for acquisition of CCR7 and for expression of CD107a [38].

**Figure 3:**
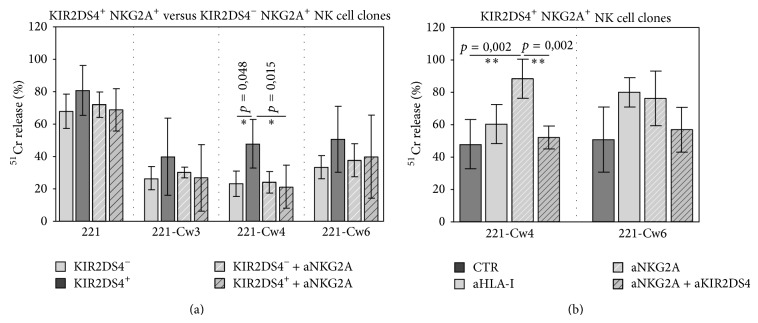
Comparison between KIR2DS4^+^ and KIR2DS4^−^ NK cell clones in the killing of transfected 221 cell lines. (a, b) The cytolytic activity of KIR2DS4^−^ NKG2A^+^ and KIR2DS4^+^ NKG2A^+^ NK cell clones (derived from a B haplotype donor) was analyzed against untransfected or Cw3-, Cw4-, or Cw6-transfected 221 cells in the absence or in the presence of mAbs to the indicated molecules. The *E*/*T* ratio used was 1 : 1. The average of 4 independent experiments and standard deviation (mean ± SD) are indicated. ^∗∗^ *p* < 0.01; ^∗^ *p* < 0.05.

As shown in Figure 3(a), in the absence of mAbs, killing of HLA-transfected 221 cells by KIR2DS4^+^ NKG2A^+^ NK cell clones was higher than lysis of KIR2DS4^−^ NKG2A^+^ NK cell clones, in particular against 221-Cw4. Moreover, mAb-mediated masking of KIR2DS4 alone induced a significant decrease of killing against 221-Cw4 cells.

Notably, the cytolytic activity of both types of NK cell clones (KIR2DS4^+^ NKG2A^+^ and KIR2DS4^−^ NKG2A^+^) against HLA-transfected 221 cells was lower as compared to that against untransfected target cells (Figure 3(a)). As expected, this poor cytotoxicity depended on inhibition due to the NKG2A-mediated recognition of HLA-E molecules on transfected target cells. Indeed, mAb-mediated masking of NKG2A reconstituted lysis (Figure 3(b) and data not shown). The further addition of anti-KIR2DS4 mAb significantly decreased the reconstituted lysis against Cw4-trasfectants but only with KIR2DS4^+^ NK cell clones (Figure 3(b)).

Altogether these data confirm that KIR2DS4 is able to recognize HLA-Cw4 molecules. Indeed, a significant inhibition of cytotoxicity against Cw4-transfected target cells could be detected not only by the use of anti-KIR2DS4 alone but also by the combined masking of KIR2DS4 and NKG2A.

Our study demonstrates that KIR2DS4^+^ NKG2A^+^ NK cell clones may take up CCR7 molecules upon interaction with CCR7^+^ cells expressing specific HLA class I alleles (including HLA-Cw4) and that KIR2DS4 exerts a key role in this process as demonstrated by inhibition of CCR7 uptake upon its mAb-mediated masking. This inhibition could be more detectable by blocking with specific antibodies the inhibitory interactions of NKG2A molecules with their ligands (HLA-E), whose expression is induced on 221 cells by HLA-C1 or -C2 alleles [4].

The finding that the activating signal delivered by KIR2DS4 is not always sufficient to overcome the signals mediated by NKG2A receptors could be due at least in part to a higher expression of this receptor on some of the clones analyzed.

In addition, also thanks to the use of novel reagents that until very recently were not available, we have been able to accurately characterize the phenotype of NK cell clones and analyze the specificity of the KIR2DS4 receptor. In particular we show that, in addition to HLA-Cw4, KIR2DS4 can recognize HLA-Cw6 alleles, even if more weakly, and that also this interaction allows the uptake of CCR7 (in the absence of inhibitory signals).

The finding that, as previously shown, the* de novo* expression of CCR7 enables NK cells to be redirected from inflamed peripheral tissues to secondary lymphoid organs confers to these data important implications both in anti-tumor responses and in allogeneic HCT [36, 38, 53–55].

The CCR7 expression can be induced on NK cells that normally do not express this receptor also in the presence of the proinflammatory cytokine IL-18 [44]. However, it is interesting to note that KIR2DS4^+^ NK cell clones were unable to acquire CCR7 in an IL-18 dependent fashion, since, different from resting NK cells, IL-2 activated NK cells (including NK cell clones) express very low level of IL-18R*α*.

These data suggest that the uptake of CCR7 by activated NK cells may be closely dependent on the mechanism of trogocytosis.

In line with previous reports [15, 16], KIR2DS4 receptor plays a prominent role also in the NK-mediated killing of Cw4-transfected 221 cell lines since its blocking with specific mAbs could significantly decrease this lysis.

Thus, in given donor/recipient pairs, KIR2DS4 expression may contribute to potentiating NK cell function by increasing both the cytotoxicity and the expression of CCR7 on their surface. This last event has important implications during haploidentical HCT in which the migration of alloreactive NK cells to lymph nodes may be crucial to prevent the GvHD and HvG, by killing recipient DCs and T cell blasts directly in this compartment, and to increase the success of transplantation.

## 4. Conclusions

The activating NK receptor KIR2DS4 can modify some phenotypic and functional features of NK cells. In particular, we showed that:

1. By interacting with CCR7^+^ target cells expressing HLA-Cw4 (or HLA-Cw6) alleles, KIR2DS4 is capable of inducing the uptake of CCR7 by KIR2DS4^+^ NKG2A^+^ NK cell clones. This event is, at least in part, regulated by inhibitory signals generated via NKG2A.
2. The* de novo* CCR7 expression by KIR2DS4^+^ NK cell clones is dependent on trogocytosis, whereas it cannot be mediated by IL-18 (known to induce CCR7 expression on CD56^dull^ NK cell subset) since activated NK cells express low level of IL-18R*α*.
3. The expression of KIR2DS4 by NKG2A^+^ NK cell clones represents an advantage in the recognition and killing of target cells expressing HLA-Cw4 alleles.
